# Coronary Artery Bypass Grafting: A Comparative Exercise between Patients from the BYPASS Registry and Patients from a University Hospital

**DOI:** 10.21470/1678-9741-2022-0026

**Published:** 2023-07-18

**Authors:** Marcela Accari de Almeida, Livia Arcêncio, Gabriel B Giuliani, Omero Benedicto Poli Neto, Alfredo José Rodrigues, Walter Vilella de Andrade Vicente, Paulo Roberto Barbosa Évora

**Affiliations:** 1 Hospital das Clínicas da Faculdade de Medicina de Ribeirão Preto da Universidade de São Paulo (HCFMRP-USP), Ribeirão Preto, São Paulo, Brazil

**Keywords:** Coronary Artery Bypass, Postoperative Care, Registries, Electronics, Treatment Outcome

## Abstract

**Introduction:**

The coronary artery bypass grafting (CABG) data provided by the Brazilian Registry of Cardiovascular Surgeries in Adults (BYPASS) Registry is a Brazilian reality.

**Objective:**

To carry out a comparative exercise between the BYPASS Registry published data and data from patients operated on in a randomly chosen period (2013-2015) at Hospital das Clínicas da Faculdade de Medicina de Ribeirão Preto da Universidade de São Paulo (HCFMRP-USP).

**Methods:**

This is a retrospective study reviewing 173 electronic medical records of CABG patients from the HCFMRP-USP. These data were compared with the BYPASS Registry published data. Chi-square test was used to verify the changes within the prevalence of adequate/inadequate biochemical tests before and after surgery. The sample was divided into groups consistent with cardiopulmonary bypass (CPB) time (CPB ≤ 120 minutes and CPB > 120 minutes). For the complications, prevalence by the chi-square test was adopted. Significant P-values are < 0.05.

**Results:**

The comparative operative data of the BYPASS Registry and the HCFMRP-USP patients were quite similar, except for the isolate use of only arterial grafts, which was more frequent on HCFMRP-USP patients (30.8% vs. 15.9%), and the use of radial artery, also more frequent on HCFMRP-USP patients (48.8% vs. 1.1%)

**Conclusion:**

The comparative study suggested that the BYPASS Registry should be a reference for CABG quality control.

## INTRODUCTION

**Table:** 

Abbreviations, Acronyms & Symbols				
AMI	= Acute myocardial infarction		HF	= Heart failure
ARDS	= Acute respiratory distress syndrome		INR	= International normalized ratio
ASA	= Acetylsalicylic acid		ITA	= Internal thoracic artery
BJCVS	= Brazilian Journal of Cardiovascular Surgery		LITA	= Left internal thoracic artery
BYPASS	= Brazilian Registry of Cardiovascular Surgeries in Adults		MV	= Mechanical ventilation
CABG	= Coronary artery bypass grafting		NYHA	= New York Heart Association
COPD	= Chronic obstructive pulmonary disease		PCI	= Percutaneous coronary intervention
CPB	= Cardiopulmonary bypass		PTCA	= Percutaneous transluminal coronary angioplasty
EF	= Ejection fraction		RITA	= Right internal thoracic artery
GOT	= Glutamic-oxaloacetic transaminase		SVG	= Saphenous vein graft
GPT	= Glutamic-pyruvic transaminase			
HCFMRP-USP	= Hospital das Clínicas da Faculdade de Medicina de Ribeirão Preto da Universidade de São Paulo			

Worldwide, cardiovascular disease remains the leading cause of death for both women and men. Regulatory agencies and public funding agencies have put forth recommendations to improve inclusivity and diversity in clinical trials; however, only limited progress has been made. According to Khan & Mitchell (2021)^[1]^, the homogeneity of cardiovascular clinical trial populations limits the generalizability of results and compounds health inequities faced by women, older adults, and people of color. This article highlights the importance of diversity in clinical trial populations and describes multifaceted interventions that might help to close the diversity gap in trial enrolment. Although it has high international prestige, Brazilian cardiac surgery failed to carry out a large “trial” on coronary artery bypass grafting (CABG)^[2]^.

The Brazilian Registry of Cardiovascular Surgeries in Adults (BYPASS) Registry project is fulfilling the purpose of portraying cardiovascular surgery in Brazil^[3,4]^ and should be a crucial reference for indications and comparisons of therapeutic procedures. Therefore, this presentation aimed to carry out a comparative exercise between BYPASS Registry data and data from patients operated on in a randomly chosen period (2013-2015) at Hospital das Clínicas da Faculdade de Medicina de Ribeirão Preto da Universidade de São Paulo (HCFMRP-USP). This presentation intends to be a kind of test incentive and consequently reinforces the BYPASS Registry as a pivotal Brazilian cardiac surgery database.

## METHODS

### Patients

*The HCFMRP-USP group* - A retrospective study was conducted by reviewing 173 medical records of patients submitted to myocardial revascularization surgery at the HCFMRP-USP. Data were collected and analyzed after approval by the Research ethics panel of the hospital (HCRP process No. 7223" 2017). Eighteen patients have been excluded from the study due to scarcity of data.

*The BYPASS Registry group* - Data were obtained with permission from the Brazilian Journal of Cardiovascular Surgery (BJCVS).

Routine biochemical tests (creatinine, urea, direct and total bilirubin, albumin, international normalized ratio (INR), troponin, creatine phosphokinase, creatine kinase-myocardial band, alkaline phosphatase, gamma-glutamyl transferase, glutamic-oxaloacetic transaminase [GOT], and glutamic-pyruvic transaminase [GPT]) were evaluated. Surgery data (such as time of surgery, cardiopulmonary bypass [CPB], and ischemia) also were collected because they got to use vasoactive drugs after CPB.

Postoperative parameters were also evaluated, as the necessity for blood transfusion and intra-aortic balloon use, the prevalence of deaths and causes, and the need for reintervention and their reasons.

### Statistical Analysis

Continuous variables were presented as mean ± standard deviation and categorical variables as percentages. The chi-square test was used to verify the changes within the prevalence of adequate/inadequate biochemical tests before and after surgery. The sample was divided into groups consistent with CPB time (CPB ≤ 120 minutes and CPB > 120 minutes) and mechanical ventilation (MV) time (MV ≤ 2 days and MV > 2 days). For the complications, prevalence by the chi-square test was adopted. The collected data was analyzed using the IBM Corp. Released 2011, IBM SPSS Statistics for Windows, version 20.0, Armonk, NY: IBM Corp. Significant *P*-values are < 0.05.

## RESULTS

### Patients Operated at HCFMRP-USP

1) The prevalence of patients undergoing CABG was higher in males; 2) most had hypertension, severe coronary lesion, dyslipidemia, and were smokers; 3) the foremost commonly used medications were acetylsalicylic acid (ASA), beta-blockers, and simvastatin; 4) there was evidence of renal and hepatic dysfunction; 5) most of the surgical reinterventions were bleeding, stroke, and acute myocardial infarction (AMI). Numbers are presented in Table 1.

**Table 1:** Clinical-epidemiological characteristics of the patients (n = 173).

	Number of patients	Percentage (%)
*Gender*		
Male	155	89.6
Female	18	10.4
*HF*		
Yes	48	27.7
No	125	72.3
*NYHA*		
I	33	19
II	43	24.9
III	72	41.6
IV	25	14.5
*Severe coronary artery disease*		
Yes	139	80.3
No	34	19.7
*Hypertension*		
Yes	161	93.1
No	12	6.9
*Diabetes mellitus*		
Yes	94	54.3
No	79	45.7
*Obesity*		
Yes	50	28.9
No	123	71.1
*Dyslipidemia*		
Yes	138	79.8
No	35	20.2
*Chronic kidney disease*		
Yes	8	4.6
No	165	95.4
*Smoking*		
Yes	86	49.7
No	87	50.3
*Surgery*		
Urgency	28	16.2
Elective	145	83.8
*Previous CABG*		
Yes	5	2.9
Not	168	97.1
*Previous PTCA*		
Yes	25	14.5
Not	148	85.5
*Previous cardiac surgery*		
Yes	1	0.6
Not	172	99.4

CABG=coronary artery bypass grafting; HF=heart failure; NYHA=New York Heart Association; PTCA=percutaneous transluminal coronary angioplasty

Regarding renal function (assessed by creatinine and urea), most patients had good values before surgery; 86.7% of the patients had good creatinine values, while 98.3% had good urea values. However, the percentage of patients with renal dysfunction increased after surgery (*P*<0.001). Biochemical indicators related to liver function were standard in most patients before surgery. Still, it is noteworthy the increase in the number of patients with high concentrations of GOT and GPT after surgery (*P*<0.001). Albumin concentrations was adequate in all patients evaluated at both periods. Regarding the INR, 98.3% of patients were classified as acceptable before surgery, which decreased to 64.7% after it (*P*<0.001). Analysis of troponin showed that 75% of patients had values above the normality of surgery, a percentage that decreased after the surgical procedure (*P*=0.004).

Almost half of the individuals (48%) required vasoactive amines for CPB, which were dobutamine (72.3%), noradrenaline (1.2%), and a combination of both (26.5%). Of the patients requiring associated surgery (11%), 15.8% corresponded to valve replacement and 84.2% to pacemaker placement. Still, the number of patients with previous surgery was small; 2.9% had undergone previous CABG, 14.5% had prior percutaneous revascularization, and 0.6% had other cardiac surgery. For those who had undergone previous surgery, the mean CPB time was 103.6±32.9 minutes, and the aortic occlusion time was 76.9±25.7 minutes.

### Patients From the Bypass Registry

Data of the BYPASS Registry group were obtained from the BJCVS.

### BYPASS Registry Data *vs.* HCFMRP-USP Data

Table 2 presents the demographic and clinical data of the cohort of patients operated on at the HCFMRP-USP in comparison with the recent results presented by the BYPASS study. Note that the results are similar. The two major causes of death were AMI and pneumonia, both with a percentage of 30.8%.

**Table 2:** Comparative general data of the BYPASS Registry and the HCFMRP-USP patients.

	BYPASS Registry Total (n=2292)	HCFMRP-USP Total (n=173)	*P*-value
Sex (female)	665/2292 (29%)	18/173 (10.4%)	< 0.0001
Diabetes	973/2292 (42.5%)	94/173 (54.3%)	0.0031
Dyslipidemia	1250/2292 (54.5%)	138/173 (79.8%)	< 0.0001
Infarction	941/2292 (41.1%)	6/173 (3.4%)	< 0.0001
Heart failure	328/2292 (14.3%)	48/173 (27.7%)	< 0.0001
NYHA (2)	181/312 (58%)	43/173 (24.9%)	< 0.0001
NYHA (3)	82/312 (26.3%)	72/173 (41.6%)	0.0007
NYHA (4)	7/312 (2.2%)	25/173 (14.5%)	< 0.0001
Kidney failure	111/2292 (4.8%)	43/173 (24.85%)	< 0.0001
Current smoker	309/2292 (13.5%)	86/173 (49.7%)	< 0.0001
Ex-smoker	570/2286 (24.9%)	23/173 (13.29%)	0.0008
Pacemaker	20/2292 (0.9%)	16/173 (9.24%)	< 0.0001
COPD	128/2292 (5.6%)	28/173 (16.18%)	< 0.0001
EF < 40%	185/1904 (9.7%)	40/173 (23.12%)	< 0.0001

COPD=chronic obstructive pulmonary disease; EF=ejection fraction; HCFMRP-USP=Hospital das Clínicas da Faculdade de Medicina de Ribeirão Preto da Universidade de São Paulo; NYHA=New York Heart Association

Thirty days after surgery, analyzing the data from the patients’ records regarding hospital evolution and outpatient returns, it was observed that most patients (68.2%) had no signs, symptoms, or comorbidities, suggesting an excellent surgical evolution; 15% of the patients had symptoms, and the most prevalent was dyspnea (7.5% of the study patients)

The comparative operative data of the BYPASS Registry and the HCFMRP-USP patients also were quite similar, except for the use of only arterial grafts, which were more frequent on HCFMRP-USP patients (30.8% *vs.* 15.9%); radial artery was also more used on the HCFMRP-USP patients (48.8% *vs.* 1.1%). Intraoperatively, almost half of the patients required vasoactive drugs to undergo CPB, and the primary drug used was dobutamine (72.3%). Although only 11% had any associated cardiac surgery, 84.2% of these were pacemaker placement (Tables 3 and 4).

**Table 3:** Comparative operative complications data of the BYPASS Registry and the HCFMRP-USP patients.

	BYPASS Registry Total (n=2292)	HCFMRP-USP Total (n=173)	*P*-value
Myocardial infarction	11/2292 (0.5%)	4/173 (2.31%)	0.0131
Major bleeding	119/2292 (5.2%)	4/173 (2.31%)	0.1345
Transfusion	539/2292 (23.5%)	41/173 (23.7%)	0.9564
Postperfusion syndrome	15/2292 (0.7%)	1/173 (0.5%)	0.9039
Arrhythmia	70/2292 (3.1%)	8/173 (4.6%)	0.3615
Low cardiac output	102/2292 (4.5%)	8/173 (4.6%)	0.9149
Use of vasoconstrictors	1137/2292 (49.6%)	83/173 (48%)	0.7378
Intraoperative death	12/2292 (0.5%)	1/173 (0.6%)	0.9240

HCFMRP-USP=Hospital das Clínicas da Faculdade de Medicina de Ribeirão Preto da Universidade de São Paulo

**Table 4:** Comparative operative data of the BYPASS Registry and the HCFMRP-USP patients.

	BYPASS Registry Total (n=2292)	HCFMRP-USP Total (n=173)	*P*-value
Open-heart surgery	2291/2292 (99.95%)	173/173 (100%)	0.7835
Minimally invasive surgery	1/2292 (0%)	0/173 (0%)	0.7835
Use of CPB	1994/2292 (87%)	-	-
Cardioplegia	1899/1994 (95.2%)	-	-
Grafts			
SVG	1925/2289 (84.1%)	111/172 (64.5%)	< 0.0001
No SVG (only arterial grafts)	364/2289 (15.9%)	53/172 (30.8%)	< 0.0001
Only SVG	176/2289 (7.7%)	8/172 (4.6%)	0.1900
Use of LITA	2083/2289 (91%)	149/172 (86.6%)	0.0771
Use of RITA	129/2289 (5.6%)	12/172 (6.9%)	0.5756
One ITA			
Use of LITA or RITA	1994/2289 (87.1%)	139/172 (80.8%)	0.0259
Use of only LITA or RITA	300/2289 (13.1%)	14/172 (8.13%)	0.0259
Two ITA			
Use of LITA and RITA	109/2289 (4.8%)	11/172 (6.39%)	0.4379
Use of only LITA and RITA	48/2289 (2.1%)	0/172 (0%)	0.1027

CPB=cardiopulmonary bypass; HCFMRP-USP=Hospital das Clínicas da Faculdade de Medicina de Ribeirão Preto da Universidade de São Paulo; ITA=internal thoracic artery; LITA=left internal thoracic artery; RITA=right internal thoracic artery; SVG=saphenous vein graft

Concerning comparative postoperative data, the BYPASS Registry patients presented low reoperation rate (2.3 *vs.* 8%), less renal failure (4.8 *vs.* 24.4%), and low mortality (2.8 *vs.* 7.3%); the HCFMRP-USP patients presented fewer arrhythmias (4.6 *vs.* 14.1%)

Finally, the other postoperative differences between BYPASS Registry and HCFMRP-USP patients are shown on Table 5.

**Table 5:** Comparative postoperative data of the BYPASS Registry and the HCFMRP-USP patients.

	BYPASS Registry Total (n=2292)	HCFMRP-USP Total (n=173)	*P*-value
Reoperation	52/2280 (2.3%)	14/173 (8%)	< 0.0001
Major bleeding	62/2280 (2.7%)	4/173 (2.3%)	0.9399
PCI	5/2280 (0.2%)	-	-
Mechanical ventilation > 24 h	ex120/2280 (5.3%)	12/173 (6.9%)	0.4439
Tracheostomy	12/2280 (0.5%)	1/173 (0.5%)	0.9280
ARDS	33/2280 (1.4%)	-	-
Low cardiac output	73/2280 (3.2%)	8/173 (4.6%)	0.4302
Kidney failure	84/2280 (3.7%)	27/173 (15.6%)	< 0.0001
Dialysis	30/84 (35.7%)	-	-
Coagulopathy	23/2280 (1%)	-	-
Transfusion	446/2280 (19.6%)	41/173 (23.7%)	0.2238
Arrythmias	336/2280 (14.7%)	8/173 (4.6%)	0.0003
Need for pacemaker	91/2280 (4%)	-	-
Infection	118/2280 (5.2%)	8/173 (4.6%)	0.8902
Myocardial infarction	27/2280 (1.2%)	4/173 (2.3%)	0.3537
Vasoplegic syndrome	27/2280 (1.2%)	1/173 (0.5%)	0.7245
Mortality	64/2292 (2.8%)	13/173 (7.5%)	0.0013
Heart failure	29/2280 (1.3%)		-

ARDS=acute respiratory distress syndrome; HCFMRP-USP=Hospital das Clínicas da Faculdade de Medicina de Ribeirão Preto da Universidade de São Paulo; PCI=percutaneous coronary intervention

**Fig. 1:**
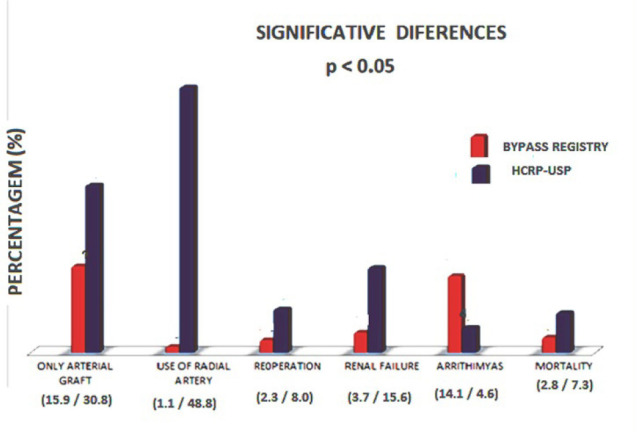
Representative differences between BYPASS Registry and Hospital das Clínicas da Faculdade de Medicina de Ribeirão Preto da Universidade de São Paulo (HCFMRP-USP) patients.

## DISCUSSION

The BYPASS Registry database is an important reference for indications and comparisons of therapeutic procedures, as well as to propose subsequent models to improve patient safety and the quality of surgical practice in the country^[3-5]^.

Concerning the most important differences, the BYPASS Registry patients were older, and presented more heart failure. It is notable the AMI difference, which was more prevalent in the BYPASS Registry (41.1%) than in the HCFMRP-USP (3.4%) patients. Also, it is notable that operative mortality in the BYPASS Registry (2.8%) was lowest than HCFMRP-USP 30-day mortality (7.5%). The postoperative hospital outcomes were analyzed. Patients referred to CABG in Brazil are predominantly male (71%), with prior myocardial infarction in 41.1% of cases, diabetes in 42.5%, and ejection fraction < 40% and > 9.7%. The Heart Team surgery decided 32.9% of the surgical indications. Most of the patients underwent CPB (87%), and cardioplegia was the strategy of myocardial protection chosen in 95.2% of the cases. The left internal thoracic artery was used as a graft in 91% of the cases, the right internal thoracic artery in 5.6%, and the radial artery in 1.1%. The saphenous vein graft was used in 84.1% of the patients, being the only graft employed in 7.7% of the patients. The median number of coronary vessels treated was three. Operative mortality was 2.8%, and the incidence of cerebrovascular accident was 1.2%. The HCFMRP-USP postoperative 30-day hospital mortality rate value is higher than the BYPASS Registry value, but it is an acceptable value, according to the present literature, even though the study was done in a university hospital, where the work involves the participation of academics and residents, as well as teachers and bosses.

The analysis of medications in preoperative use was complicated by the generality of drugs used in the clinics where the patients came from. The more frequent medications were beta-blockers, vasodilators, calcium channel blockers, alpha-2-adrenergics, diuretics, angiotensin receptor blockers, angiotensin-converting enzyme inhibitors, digitalis, anti-arrhythmic alpha-blockers, antiplatelet agents, statins and other drugs used in dyslipidemia, insulin, oral hypoglycemic agents, pump inhibitor protons, and levothyroxine. The most prevalent drug was ASA, which was used by 89.6% of patients. Finally, to speculate about high mortality among different cohorts, it is pertinent to discuss briefly at least two possibly related factors: cardiac specialty hospitals and low-volume hospitals.

An analysis of the database of the Brazilian Universal Healthcare System, published in 2006, revealed that from 2000 to 2003, 115 patients underwent cardiac surgery with an overall hospital mortality of 8%. The hospital mortality was 6.1% for congenital heart surgery, 7% for coronary artery bypass, 8.9% for heart valve surgery, and 16.5% for “complex” operations (*e.g.*, thoracic aorta and combined procedures). The investigators also found that hospitals with annual volumes of less than 341 operations had higher mortality. Unfortunately, it was not possible to define this parameter in the present study, since a group of patients were operated on in a single hospital (HCFMRP-USP) and BYPASS Registry patients were operated on in several hospitals. But the anecdotal observation remains that mortality is higher in services that operate less. It would still be necessary to consider a bias created by the possibility of centralizing surgeries to correct complex heart diseases for specialized hospitals with fewer surgeries.

According to Cram (2005)^[6]^ and Hwang (2007)^[7]^, it is supposed that cardiac specialty hospitals assert better patient outcomes and efficiency, whereas general hospitals attract healthier patients. Favorable patient selection may occur at cardiac specialty hospitals. Although healthier patients are comparably across types of hospitals, patients with greater comorbid disease seem to experience worse 30-day post-discharge mortality at specialty hospitals^[7]^.

According to Anglemyer et al. (2014)^[8]^, researchers and organizations often use evidence from randomized controlled trials to determine the efficacy of a treatment or intervention under ideal conditions. Studies of observational designs are often used to measure the effectiveness of an intervention in “real world” scenarios. Also, according to Black (1996)^[9]^, when trials cannot be conducted, well designed observational methods offer an alternative to doing nothing. They also offer the opportunity to establish high external validity, something that is difficult to achieve in randomized trials. Instead of advocates of each approach criticizing the other method, everyone should pursue for greater rigor in the in the execution of research, regardless of the method used.

### Limitations

The BJCVS announced the fully operational BYPASS Registry in 2016, and the data inclusion has exceeded 1,500 patients in the first nine months of operation. The establishment of the BYPASS Registry sets a long-standing need for fundamental understanding of the real figures pertaining to the cardiovascular surgery practice, resulting in developing strategies for improvements in quality and excellence, the main motivation of the present investigation^[3-5]^.

## CONCLUSION

In conclusion, we emphasize three main points:

CABG data in Brazil provided by the BYPASS Registry analysis are representative of our national reality and practice. This database constitutes an important reference for indications and comparisons of therapeutic procedures, as well as to propose subsequent models to improve patient safety and the quality of surgical practice in the country.

The comparative operative data of the BYPASS Registry and the HCFMRP-USP patients were quite similar, except for the use of only arterial grafts, which were more frequent on the HCFMRP-USP patients (30.8% *vs.* 15.9%), and the use of radial artery, also more frequent on the HCFMRP-USP patients (48.8% *vs.* 1.1%)

Concerning comparative postoperative data, the BYPASS Registry patients presented low reoperation rate (2.3 *vs.* 8%), less renal failure (4.8 *vs.* 24.4%), and low mortality (2.8 *vs.* 7.3%); and the HCFMRP-USP patients presented fewer arrhythmias (4.6 *vs.* 14.1%).
